# Anxiety among caregivers of children with epilepsy from western China: A cross-sectional survey

**DOI:** 10.1097/MD.0000000000019237

**Published:** 2020-02-21

**Authors:** Chunsong Yang, BingYao Kang, Yifei Mao, Qunfen Xu, Dan Yu, Lingli Zhang

**Affiliations:** a Department of Pharmacy, Evidence-based Pharmacy Center, West China Second Hospital, Sichuan University; b Key Laboratory of Birth Defects and Related Diseases of Women and Children (Sichuan University), Ministry of Education, China; c Department of Epidemiology, West China School of Public Health, and West China Fourth Hospital; d Department of Pediatric Clinic, West China Second Hospital; e West China School of Pharmacy; f Department of Children's Genetic Endocrinology and Metabolism, West China Second Hospital, Sichuan University, China.

**Keywords:** anxiety, caregivers, children, epilepsy

## Abstract

The current study aimed to evaluate the status of anxiety among caregivers of children with epilepsy and examine the associated factors.

A cross-sectional study was conducted in western China, which consecutively recruited children with epilepsy in 2018. The self-rating anxiety scale (SAS) was used to assess the status of anxiety among caregivers of children with epilepsy. We collected information about aspects of sociodemographic data, disease status, attitude of caregivers towards the disease and family conditions as independent variables, using multiple linear regression to analyze factors related to the status of anxiety among caregivers.

A total of 334 participants were included in the study with a response rate of 95.4% (334/350). The mean age of children with epilepsy was 6.05 ± 4.11 years. 7.2% (24/334) of patients were newly diagnosed and 55.4% (185/334) of patients presented with generalized epilepsy. A total of 25.7% (86/334) of caregivers among children with epilepsy presented the symptom of anxiety, with the scores of SAS 44.31 ± 10.558. SAS scores were negatively correlated with the children's age (*B* = −0.141; standard error = 0.135; *P* = .008), attitude towards seizures (*B* = −0.153; standard error = 1.192; *P* = .004) and medical expenses payment (*B* = −0.169; standard error = 1.703; *P* = .002).

Symptoms of anxiety are common among caregivers of children with epilepsy in western China. Healthcare providers should pay more attention to caregivers with younger children, difficult financial situation, and greater fear of seizures. Exploring mental health interventions for caregivers is important.

## 1 Introduction

Epilepsy is one of common neurological disorders caused by paroxysmal abnormal discharge of brain neurons for a range of reasons, characterized by epileptic seizures.^[1,2]^ The prevalence rate of epilepsy around the world is 6‰ to 7‰, with approximately 70 million patients suffered from epilepsy.^[2–4]^ Among children, the lifetime rate of epilepsy is 1%, and the time-point prevalence rate is 6.3‰.^[5]^ In the United States, 2.5 million people, including 3,25,000 children under the age of 15, suffer from epilepsy.^[6]^ In China, the prevalence rate of epilepsy is 1.43‰ to 7.98‰, and the prevalence rate among children is 3.9‰ to 5.1‰.^[7,8]^ The influences of epilepsy on children and their caregivers are diverse, with medical, psychological, educational, personal, economic, and social effects.^[5]^

Anxiety is more frequent among people with epilepsy and their caregivers compared with the general population, at all ages.^[9]^ Anxiety disorders not only affect a person's emotional state, but may also cause physical symptoms such as increasing heart rate and tremors. Several previous studies have evaluated the status of anxiety among parents of children with epilepsy. In 2016, a systematic review^[10]^ was conducted to focus on symptoms of anxiety among parents of children with epilepsy, including 15 studies involving 1434 respondents. The results revealed that 9% to 58% of parents exhibited symptoms of anxiety, but the possible correlates of parental anxiety in childhood epilepsy that were considered varied widely across studies.^[10]^ In addition, 3 of the studies included in the review were conducted in Beijing, China, and the factors affecting anxiety were inconsistent.^[10]^ To the best of our knowledge, no studies of populations in west China have been published. Therefore, the current study sought to:

(1) assess anxiety among caregivers of children with epilepsy from western China; and
(2) identify patient, medication, caregiver, and environment-related characteristics associated with anxiety among caregivers.

## 2 Methods

### 2.1 Setting

This cross-sectional study was conducted at West China Second Hospital, Sichuan University. The Hospital was located in the city of Chengdu, Sichuan Province, China, and is the largest and most authoritative center for the diagnosis and treatment of pediatric patients in western China.

### 2.2 Study design and sample selection

The participants in this study were examined from January to May in 2018 in the pediatric neurology clinic in West China Second Hospital. The inclusion criteria were:

(1) the child was less than 18 years old;
(2) epilepsy was clinically diagnosed according to the International League Against Epilepsy (ILAE) 2014 criteria;
(3) the caregiver of the child with epilepsy was willing to participate in this study.

The exclusion criteria were:

(1) other chronic diseases in the child with epilepsy, which could affect the emotional states of caregivers (i.e., congenital heart disease, diabetes);
(2) the caregiver was illiterate; and
(3) lack of consent.

### 2.3 Data collection

A questionnaire was designed by a trained physician and pharmacist and divided into 2 sections. The first section was focused on the characteristics of children with epilepsy, including:

(1) children's basic characteristics (name, age, place of residence);
(2) disease status (newly diagnosed patient or not, seizure type, family history of epilepsy, comorbidity, seizure frequency, regular review); and
(3) medication status (quantity of medication, adverse reactions, time for medication use).

The second section was focused on the caregivers, including:

(1) sociodemographic characteristics (caregivers, age of caregivers, caregivers’ education level, caregivers’ working status);
(2) treatment attitude (caregivers’ attitude towards seizures, confidence in treatment);
(3) environmental factors (parents’ marital status, total household income, medical expenses payment).

### 2.4 Instruments

The self-rating anxiety scale (SAS) was used to evaluate the symptoms anxiety of the caregivers. The SAS is reported to have good reliability and validity in the Chinese population.^[10]^ The SAS scale has 20 items and uses a 4-point scoring system to measure the frequency of symptoms (1: no or a little time, 2: a small part of the time, 3: a considerable amount of time, 4: most or all of the time). Of these, 15 items use negative words, which are scored according to the above method from 1 to 4, while the other 5 statements use positive words, which are scored from 4 to 1. Adding the scores of all items produces the rough score, and multiplying the score by 1.25 produces the standard score. The standard score on the SAS is graded according to the results of the scores (normal: <50 points; anxiety: >50 points).

### 2.5 Data analysis

Data analysis was conducted after data collection and initial processing in 2 parts.

First, quantitative data were expressed as mean ± SD. Second, the normality of the data was tested using the Kolmogorov–Smirnov test. To compare quantitative variables between groups, the variance analysis or the Mann–Whitney *U* test were used for the normally distributed data and non-normally distributed data, respectively.

Factors with univariate *P* ≤ 0.10 were included in the multiple linear regression model for univariate analysis. Data analysis was performed using SPSS version 22, and the threshold for statistical significance was set at *P* < .05.

### 2.6 Ethical issues

The design of the study was in accordance with the Helsinki Declaration. The study began after the approval of the Office of Research Ethics Committees of West China Second Hospital. Voluntary written informed consent was provided by all participants at enrollment in the study.

## 3 Results

### 3.1 Demographic characteristics of the patients

In total, 334 patients participated in the study, with a response rate of 95.4% (334/350). The age of children with epilepsy ranged from 0.2 to 17.8 years, with a mean age of 6.05 ± 4.11 years. Regarding the type of visit, 7.2% (24/334) of patients were newly diagnosed. Concerning seizure type, 55.4% (185/334) of patients presented with generalized epilepsy and 44.6% (149/334) of patients presented with focal/partial epilepsy. In total, 7.8% (26/334) of patients had a family history of epilepsy, and 39.2% (131/334) had comorbidities.

### 3.2 The prevalence of anxiety

A total of 25.7% (86/334) of caregivers among children with epilepsy presented with symptoms of anxiety, with a mean SAS score of 44.31 ± 10.558.

### 3.3 Factors influencing SAS score (Tables 1 and 2)

#### 3.3.1 Factors for children with epilepsy

Univariate analysis revealed that the age of the children (*P* = .003) and seizure frequency (*P* = .004) were correlated with SAS scores.

**Table 1:**
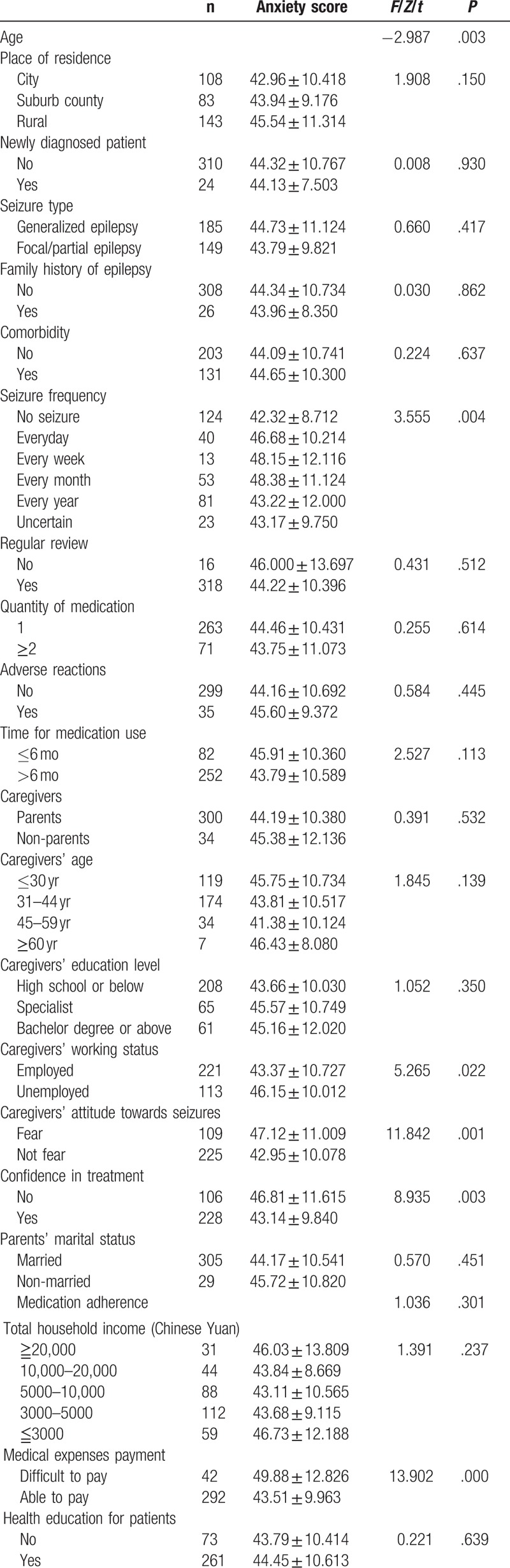
Characteristics of the study population.

**Table 2:**
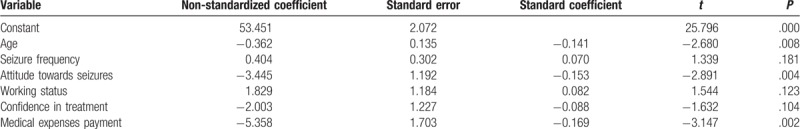
Multiple linear regression analysis of factors influencing anxiety of caregivers of children with epilepsy.

#### 3.3.2 Factors for caregivers

Univariate analysis revealed that caregivers’ working status (*P* = .022), caregivers’ attitude towards seizures (*P* = .001), confidence in treatment (*P* = .003), and medical expenses payment (*P* = .000) were correlated with SDS scores.

We included factors with univariate *P* ≤ .10 in the multiple linear regression model. The results revealed that SAS scores were negatively correlated with children's age (*B* = −0.141; standard error = 0.135; *P* = .008), attitude towards seizures (*B* = −0.153; standard error = 1.192; *P* = .004), and medical expenses payment (*B* = −0.169; standard error = 1.703; *P* = .002). Caregivers with younger children, those who were more afraid of seizures, and those who were unable to pay medical expenses were more likely to experience anxiety.

## 4 Discussion

We conducted a cross-sectional study to evaluate anxiety status among caregivers of children with epilepsy, and examined which factors were associated with anxiety. The analysis included 334 participants, revealing that 25.7% (86/334) of caregivers of children with epilepsy presented symptoms of anxiety, with an average SAS score of 44.31 ± 10.558. The current results suggest that healthcare providers should pay more attention to psycho-emotional symptoms among caregivers of children with epilepsy.

Regarding the factors influencing symptoms of anxiety for caregivers of children with epilepsy, multiple linear regression revealed that SAS scores were significantly correlated with children's age, attitude towards seizures, and medical expenses payment. There are several possible reasons for these findings:

(1) parents of younger children may worry more about their health, disease prognosis, and the impact of the disease on studying and working, causing caregivers to be more likely to experience anxiety;
(2) the attitudes of the caregivers towards seizures have an important influence on anxiety, because, regardless of the disease severity, caregivers who understand more about seizures may have more confidence about treatment;
(3) outpatient treatment is generally not publicly funded, and epilepsy treatment is long-term, so the cost of treatment represents a substantial disease burden for most families.

Being unable to afford treatment may induce anxiety among caregivers.

The prevalence of anxiety symptoms among caregivers in the current study was in accord with the range of 9% to 58% reported in a systematic review by Jones and Reilly.^[11]^ However, the influential factors identified in the current study differed from those reported in some previous studies, such as Yong et al,^[12]^ who found that parental anxiety was significantly correlated with children's seizure frequency, average monthly family income per person, and parents’ knowledge about epilepsy (*P* = .000). In addition, Chapieski et al^[13]^ reported that higher levels of maternal anxiety were associated with lower socioeconomic status, more family stress, and higher generalized anxiety. However, the factor of family income was likely to be related to the factor of medical expenses payment, because people with lower incomes would be expected to have more difficulty paying for medical expenses. Williams et al^[14]^ reported that parental anxiety did not differ by seizure type or level of seizure control, in accord with the current results.

The current study involved several limitations, including:

(1) participants were from hospital-based samples, which may not represent the whole population of children with epilepsy;
(2) this study used a cross-sectional study design, which only allows for conclusions regarding correlation, not causation;
(3) caregivers who visit the hospital may have relatively higher economic status than the general population of caregivers, possibly paying more attention to the disease and experiencing a higher level of anxiety.

Further studies should be conducted to overcome these shortcomings.

## 5 Conclusions

Symptoms of anxiety are common among caregivers of children with epilepsy in western China. Healthcare providers should pay more attention to caregivers with younger children, difficult financial situations, and greater fear of seizures. It will be important for future studies to explore mental health interventions for caregivers.

## Acknowledgments

We thank Benjamin Knight, MSc., from Liwen Bianji, Edanz Group China (www.liwenbianji.cn/ac), for editing the English text of a draft of this manuscript.

## Author contributions

**Chunsong Yang:** designed the review, collected data, carried out analysis and interpretation of the data, and wrote the review.

**BingYao Kang:** designed the review, collected data, checked the data, and wrote the review.

**Yifei Mao:** designed the review, collected data, checked the data, and wrote the review.

**Qunfen Xu:** designed the review, collected data, checked the data, and wrote the review.

**Dan Yu:** designed the review, collected data, checked the data, and wrote the review.

**Lingli Zhang:** designed the review, commented on drafts for previous version.
